# Seahorse Male Pregnancy as a Model System to Study Pregnancy, Immune Adaptations, and Environmental Effects

**DOI:** 10.3390/ijms24119712

**Published:** 2023-06-03

**Authors:** Malgorzata Kloc

**Affiliations:** 1 The Houston Methodist Research Institute, Transplant Immunology, Houston, TX 77030, USA; mkloc@houstonmethodist.org; 2 Department of Surgery, The Houston Methodist Hospital, Houston, TX 77030, USA; 3 MD Anderson Cancer Center, Department of Genetics, The University of Texas, Houston, TX 77030, USA

**Keywords:** seahorse, male pregnancy, brood pouch, placenta, MHC, pollution

## Abstract

Seahorses, together with sea dragons and pipefishes, belong to the *Syngnathidae* family of teleost fishes. Seahorses and other *Syngnathidae* species have a very peculiar feature: male pregnancy. Among different species, there is a gradation of paternal involvement in carrying for the offspring, from a simple attachment of the eggs to the skin surface, through various degrees of egg coverage by skin flaps, to the internal pregnancy within a brood pouch, which resembles mammalian uterus with the placenta. Because of the gradation of parental involvement and similarities to mammalian pregnancy, seahorses are a great model to study the evolution of pregnancy and the immunologic, metabolic, cellular, and molecular processes of pregnancy and embryo development. Seahorses are also very useful for studying the effects of pollutants and environmental changes on pregnancy, embryo development, and offspring fitness. We describe here the characteristics of seahorse male pregnancy, its regulatory mechanisms, the development of immune tolerance of the parent toward the allogeneic embryos, and the effects of environmental pollutants on pregnancy and embryo development.

## 1. What Is a Seahorse, and Why Is It Used as a Model System?

The seahorse is a fish from the *Hippocampus* genus. The name *Hippocampus* derives from the Greek word hippo, meaning ‘horse’, and campus, meaning ‘sea creature’ or ‘monster’. Seahorses, together with seadragons and pipefishes, form a family of *Syngnathidae* [1]. They are evolutionarily very old; the genus *Syngnathus* (pipefishes) is known from early Oligocene (33.9 million–28.1 million years ago) fossils [1]. Seahorses (genus *Hippocampus*) evolved from the pipefishes in the late Oligocene period [2]. Currently, there are 47 known species of seahorses. They live in shallow marine habitats, and their population is in constant decline because of pollution and overfishing for use in traditional medicine, as aquarium pets, and, in some countries, as a food delicacy. The biggest known seahorse is the big-belly (potbelly) seahorse, which reaches 35 cm in length and weighs around 35 g. The smallest are pigmy seahorses, of roughly 1.4–2.7 cm in length. One species of them, the Bargibant’s pygmy seahorses (discovered in 1969 by marine biologist George Bargibant), represents the best-camouflaged species in the world. They inhabit gorgonian coral, and their color and tubercles (nodules) covering the body (purple with pink tubercles or yellow with orange tubercles) mimic the color and shape of the host coral [3]. Like other *Syngnathidae*, seahorses undergo male pregnancy, i.e., their males carry fertilized eggs and developing embryos within a special brood pouch [4]. Depending on the species, the brood pouch forms in the abdomen, trunk, or tail. Because the pouch resembles a human uterus with a placenta, seahorses are a great model for studying various aspects of pregnancy; molecular and cellular features of embryo–parent interactions; immune tolerance; and the effects of pollutants and environmental factors on pregnancy, embryo development, and the health of progeny.

## 2. Male Pregnancy

Pregnancy is an extremely pricy investment requiring many physiological, metabolic, and anatomical changes in the parental organism, which promote all the progeny’s well-being and survival. Pregnancy has evolved independently around 150 times in different vertebrate lineages [5,6]. One of the curious evolutionary inventions is male pregnancy in seahorses and other members of *Syngnathidae* fishes. Although there is no scientific consensus on the advantages of male versus female pregnancy, one of them can be a division of reproduction costs between two parents, and another is an increase in the offspring number; while the male carries babies in the brood pouch, the mother can produce another batch of eggs.

### 2.1. Brood Pouch Development

**Figure 1:**
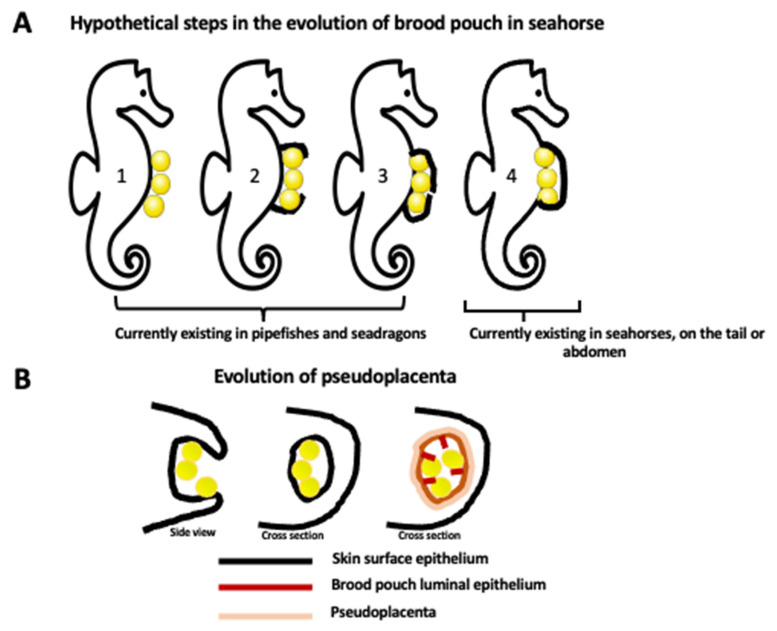
Evolution of brood pouch. (**A**) Hypothetical steps in the evolution of closed brood pouch in seahorses. Currently, pipefishes and seadragons have an external or open brood pouch (step 1, 2, 3) while seahorses have a fully closed brood pouch placed in the abdomen or in the tail (step 4). Brood pouch evolution in the *Syngnathidae* family of teleost fishes starts from the simple attachment of eggs to the surface of the skin (1) and progresses through the different degrees of enclosure of eggs by the skin flaps (2, 3), ending up with the development of a fully internal brood pouch (4). (**B**) The evolution of the internal brood pouch is accompanied by the modification of the external skin epithelium to the internal (luminal) epithelium of the pouch, and the formation of vascularized pseudoplacenta. The first drawing represents the side view, and the last two drawings represent the cross section.

**Figure 2:**
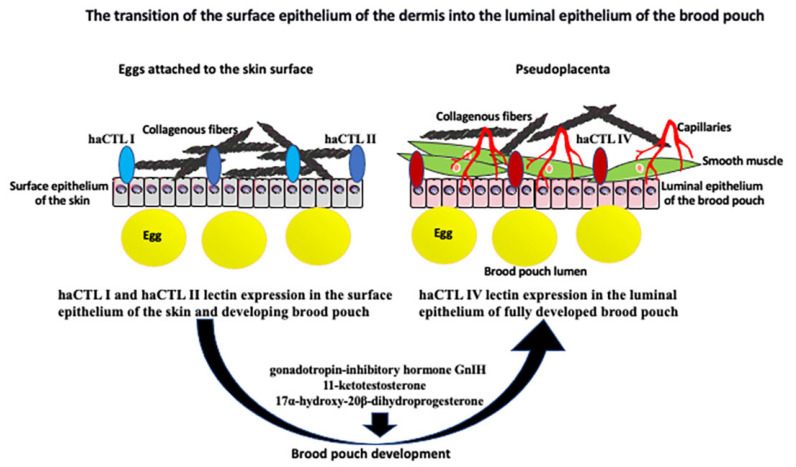
The transition of the skin surface epithelium into the luminal epithelium of the brood pouch. Surface epithelium of the skin is underlined by the collagenous fibers and expresses two forms of lectin: haCTL I and haCTL II. During development of the closed brood pouch, the external epithelium of the skin changes into the luminal (internal) epithelium of the brood pouch. Epithelial transformation correlates with the expression of haCTL IV lectin exclusively in the luminal epithelium. Epithelial transformation is accompanied by the aggregation of collagenous fibers, smooth muscle cells, and the development of capillaries below the epithelium, which all together form the pseudoplacenta that supports egg/embryo development. Development of the brood pouch and pseudoplacenta is regulated by many different nonhormonal and hormonal factors and pathways, including gonadotropin-inhibitory hormone GnIH, 11-ketotestosterone, and 17α-hydroxy-20β-dihydroprogesterone (see text for the details).

Different types of male pregnancy in various *Syngnathidae* fishes exemplify evolutionary gradation and progress in paternal involvement, from the simple attachment of eggs to the body surface to a fully developed internal pregnancy within the uterus/placenta-like pouch. In the subfamily *Nerophinae*, eggs are just attached to the skin surface without any protection. In *Oosthethus*, *Doryrhamphus*, and some *Solegnathiinae*, the attached eggs are protected by the special flaps of the skin. Finally, *Syngnathus* and *Hippocampus* have a completely closed brood pouch where developing embryos are integrated in paternal tissue and fully supplied through the vascularized placenta-like structure (Figure 1 and Figure 2) [5,7,8,9,10,11,12]. Harada et al. [8] studied in detail the brood pouch morphology and histology in five species of the *Syngnathidae* family and described five types of brood pouches. The alligator pipefish has a completely open pouch without skinfolds (type I), the messmate pipefish has an open pouch with skinfolds (type II), the seaweed pipefish has a closed pouch with skinfolds (type III), the pot-bellied seahorse has a closed pouch on the tail (type IV), and the Japanese pygmy seahorse has a closed pouch (type V) [8]. Interestingly, the type V pouch is positioned in the seahorse body cavity, and the embryos are located between the kidney and intestine [8]. The juvenile seahorse does not have a brood pouch. It develops during the post-juvenile stage [10]. While the female seahorse is laying eggs in a male brood pouch, the male fertilizes them at the pouch entry. Developing embryos are gradually enclosed within individual compartments of the placenta-like tissue of the pouch. This pseudo-placenta is vascularized and allows for the exchange of gases, nutrients, and waste removal through the epithelium that lines the pouch lumen [10]. The luminal epithelium of the pouch derives from the surface epithelium covering the seahorse’s body. Thus, at some point in pouch development, there must be a change in the epithelium properties. Kawaguchi et al. [10] studied successive stages of pouch development in the pot-bellied seahorse *Hippocampus abdominalis* as well as the molecules involved in the transition of the surface epithelium of the dermis into the luminal epithelium of the pouch. Pouch development lasts several months and starts from the long projections of the dermis (epithelium with underlying collagenous fibers) on both ventral sides of the body, which eventually fuse in the ventral midline, forming the pouch. During this stage, the pouch consists of only two layers of the dermis (epithelial layers with underlying collagenous fibers) and still lacks placenta-like features. The final step involves the formation of the pseudo-placenta. The smooth muscles and blood vessels form, and the properties of the epithelium change. The epithelium of the dermis and developing pouch expresses two types of C-lectins, haCTL I and haCTL II. In contrast, the luminal epithelium of a fully developed pouch with a pseudo-placenta specifically expresses haCTL IV lectin, which is never expressed in skin epithelium (Figure 2) [10]. By analogy to the muscle contraction during mammalian birth, the smooth muscles of the brood pouch were thought to be needed for pouch contraction during the expulsion of neonates. However, recent micro-computed tomography studies of the seahorse brood pouch showed that the muscles of the brood pouch are limited to the scattered small bundles, which cannot produce enough contraction during labor. Instead, male seahorses, in contrast to females, have large muscle bundles attached to the anal fin bones at the opening of the brood pouch, and contraction of those fin muscles, together with the body movements, opens the pouch and expels neonates [7].

### 2.2. Hormonal Regulation of Seahorse Male Pregnancy

Like in most vertebrates, reproduction stages and cycles in fishes are regulated by the hypothalamic–pituitary–gonadal (HPG) axis. In females and males, the gonadotropin-releasing hormone (GnRH) produced by the hypothalamus induces the pituitary gland to release the gonadotropins, follicle-stimulating hormone (FSH) and luteinizing hormone (LH), into the bloodstream [13], while the gonadotropin-inhibitory hormone (GnIH) inhibits gonadotropin secretion [14]. In females, gonadotropins stimulate steroid (testosterone and estradiol) production by the ovary, which, in turn, promotes secondary sexual features and sex-dependent behavior, follicle development, and vitellogenin production. Oocyte maturation is stimulated by the LH-induced production of gamete maturation-inducing steroids (MISs). Ovulation in fish is stimulated by an increase in LH followed by the release of ovarian prostaglandins, which regulate spawning behavior [15,16,17]. In males, FSH induces spermatogenesis, and LH stimulates sperm maturation, and both these gonadotropins stimulate the male gonad to produce androgens, such as testosterone and 11-ketotestosterone, which, in turn, regulate sexual dimorphism [17,18]. Gonadotropins also induce testes to produce MISs, which regulate spermiogenesis and the final maturation of spermatozoa [17,19].

Recent studies of the lined seahorse (*Hippocampus erectus*) showed that seahorses share their unique ortholog of gonadotropin-inhibitory hormone GnIH with low (35.4–65.8%) identity with the GmiH hormone of other teleost fishes. The precursor of seahorse GnIH contains LPXRFa-1 and LPXRFa-2 peptides, which are unique among vertebrates [14]. The tissue distribution analysis showed that seahorse GmIH mRNA was expressed in the hypothalamus and the brood pouch. Expression of GnIH mRNA in the hypothalamus was low during the early puberty stage and increased in the juvenile and mature seahorse. These results indicated that GnIH might be involved in the regulation of male pregnancy. Considering that GnIH negatively regulates gonadotropins, which, in turn, regulate steroid secretion, seahorse GnIH may decrease plasma testosterone levels during male pregnancy [14].

For many years, proving the existence of all the above-described hormones or their equivalents in seahorses’ blood was hindered by a minuscule volume of blood/plasma, which could be isolated from a single individual and inadequate methods/sensitivity of detection. Scobell and MacKenzie [17] extensively reviewed and tabulated all published studies on reproduction-related hormones in *Syngnathidae* male pregnancy. In the studied species of seahorses, the androgens were produced for an extended period and picked during brood pouch development and proliferation of spermatocytes. In contrast, MISs were present in seahorse circulation only before spawning. Studies of three species of *Syngnathidae* pipefish showed that depending on species, the dominant circulating androgens in breeding males are 11-ketotestosterone, testosterone, and 11β-hydroxy androstenedione [20]. Recent studies of sex steroids during pre-pregnancy, pregnancy, and post-pregnancy in the *Hippocampus erectus* seahorse showed that testosterone, 11β-hydroxytestosterone, 17α-methyltestosterone, and 17β-estradiol were higher during pre-pregnancy and diminished in post-pregnancy. In contrast, 11-ketotestosterone and 17α-hydroxy-20β-dihydroprogesterone were highest during pregnancy, which suggests their role in pregnancy regulation (Figure 2) [21]. The question remains of how steroids produced by gonads and responsible for sexual dimorphisms, such as testosterone (or ketotestosterone) in males and estrogen in females, behave during the sex-role reversal in the species with male pregnancy. Originally, researchers predicted that these species should have a reversal of sex steroids. However, this turned out to be incorrect, as the sex steroid profile of sex-role reversed and conventional species is the same. Thus, it is possible that other less studied sex steroids, such as 11β-hydroxy androstenedione (a predominant androgen in some *Syngnathidae*), are involved in the sex-role reversal process [17,20].

#### 2.2.1. Neurohypophysial Hormones

In vertebrates and some invertebrates, reproduction is also regulated by the family of peptides called the neurohypophysial hormones. These hormones are produced by specialized cells of the hypothalamus, transported inside neurosecretory granules along axons to the posterior lob (neurohypophysis or pars posterior) of the pituitary gland and eventually released into circulation. In humans, the main neurohypophysial hormones are oxytocin and vasopressin. Oxytocin regulates the contraction of the uterine smooth muscle and mammary glands, while vasopressin regulates the contraction of peripheral blood vessels, kidney function, and social behaviors such as territorial defense, aggression, and pair bonding. The main neurohypophysial hormones in birds are mesotocin and vasotocin [22]; in reptiles, oxytocin and mesotocin [23]; in amphibians, vasotocin and oxytocin [24]; and in fishes, including seahorses, vasotocin and isotocin [25]. In contrast to pairs of neurohypophysial hormones that are present in vertebrates, some invertebrates (insects, snails, and octopuses) have single neurohypophysial-like peptides [26,27].

Studies of neurohypophysial hormones vasotocin and isotocin in seahorse *Hippocampus erectus* showed that both hormones were expressed in the hypothalamus, gonads, gills, and brood pouch. Injection of vasotocin to the peritoneal cavity of pregnant male seahorses induced the release of gonadotropins (follicle-stimulating hormone and luteinizing hormone), which in turn, stimulated estrogen secretion, increased G protein-coupled estrogen receptor level in a brood pouch and caused premature birth [28]. This and earlier studies, listed in Scobell and MacKenzie [17], indicate that in male seahorses, the neurohypophysial hormones control the timing of birth via the regulation of estrogen levels.

#### 2.2.2. Prolactin

Another hormone regulated by the hypothalamus and recently shown to be involved in various aspects of male pregnancy is prolactin. Prolactin is a polypeptide hormone produced and secreted by the acidophilic lactotroph cells of the pituitary gland in response to a thyrotropin-releasing hormone released from the hypothalamus. In contrast, prolactin secretion is repressed by dopamine (produced by hypothalamus and other parts of the brain) [29,30,31]. Prolactin affects its target tissues via the prolactin receptor (PRLR) in the target cells’ membranes. In mammals, the function of prolactin is extremely broad, from electrolyte homeostasis, metabolism, growth, brain function, and immunity to the regulation of gonadal cycling, mammary gland activity, uterine functions, embryo implantation, live birth (viviparity), and parental behavior [32]. Recently, Wilson et al. [33] studied prolactin and its receptor expression in male pregnancy and analyzed how prolactin was evolutionarily co-opted to male pregnancy in *Syngnathidae* (big-belly seahorse *Hippocampus abdominalis*, greater pipefish *Syngnathus acus*, northern pipefish *S. fuscus*, and broad-nosed pipefish *S. typhle*). In contrast to many teleost fishes, which have multiple copies of PRL and PRLR, *Syngnathidae* have only a single copy (PRL1 and PRLRa) of each gene. PRL1 is expressed exclusively in the pituitary, and the expression level is stable during the whole pregnancy. This contrasts with other teleost fishes, which express PRL1 also outside of the pituitary. PRLRa is expressed in the brood pouch and all other tissues and is specifically upregulated in the male brood pouch during the second half of pregnancy [33]. Immunostaining of brood pouch tissues showed that PRL1 protein and PRLRa are present in the pouch luminal epithelium. Because the same epithelium also expressed the sodium–potassium enzyme Na+/K+ ATPase, the authors concluded that pituitary-derived PRL1 and its receptor are involved in brood pouch osmoregulation during pregnancy [33]. Previous transcriptome analysis of the seahorse brood pouch showed that Na+/K+-ATPase activity is high during the whole pregnancy [34]. The Na+/K+ ATPase has a very important role in mammalian pregnancy. It regulates ion exchange and establishes a concentration gradient across the maternal–fetal interface of the placenta, which is critical for the transfer of nutrients to the developing fetus. In most transporting epithelia, such as in the kidney and intestine, the Na+/K+ ATPase is restricted to one side of the epithelial layer, resulting in the unidirectional transfer of ions. However, in the mammalian placenta, the Na+/K+ ATPase is active on both sides of the syncytiotrophoblast layer, allowing for bidirectional ion transfer between the mother and fetus [35]. We do not know if the activity of the Na+/K+-ATPase in a seahorse pseudo-placenta is also bidirectional like in a mammalian placenta; only the functional assays of Na+/K+-ATPase activity in the pseudo-placenta of pregnant *Syngnathidae* males will provide the answer.

### 2.3. Retinoic Acid

Another molecule involved in seahorse male pregnancy regulation is retinoic acid (RA), a metabolite of vitamin A1 (all-trans-retinol). Among various retinoic acid isomers, such as 13-cis- and 9-cis-retinoic acid, the all-trans-retinoic acid (retinoic acid) is most abundant and required for growth, embryonic development, differentiation, cancer, and immunity [36,37,38,39,40,41]. During early development, RA signaling through the homeobox (Hox) and POU genes establishes the anterior–posterior axis and patterning of the embryo [39,40]. Comparative transcriptomic and metabolomic analyses of the lined seahorse *Hippocampus erectus* in different stages of brood pouch formation (unformed, newly formed, and pregnant pouch) identified 141 genes and 73 metabolites related to pouch formation and 2533 and 121 metabolites related to pregnancy. Additionally, integrative omics showed that retinoic acid (RA) synthesis and signaling were involved in brood pouch formation and seahorse pregnancy and in regulating antioxidant defenses [42]. These studies also showed that in *H. erectus* and *H. abdominalis*, RA functions upstream of testosterone and progesterone, directly regulating pouch formation via G protein-coupled receptor FSHR and cholesterol 7alpha-hydroxylase CYP7A1, a member of the cytochrome P450 superfamily of enzymes involved in the synthesis of cholesterol, steroids, and other lipids [42,43,44].

## 3. Adaptations of the Immune System to Male Pregnancy

The biggest challenge in pregnancy is how not to reject an allogeneic (nonself) embryo [45]. In mammalian pregnancy, immune tolerance toward the semi-allogenic embryo occurs through the downregulation of the major histocompatibility genes MHC I and MHC II. Paradoxically, pregnancy starts from the inflammation of the endometrium necessary for the implantation of the embryo. Chavan et al. [46] and Griffith et al. [47] called this “the inflammation paradox”. They believe that, early in evolution, acute endometrial inflammation (still occurring in some marsupials) was a natural maternal immune reaction toward the attaching embryo. Subsequently, during evolution, by suppressing the most damaging parts of the inflammatory response, the acute inflammation was transformed into the embryo-friendly process of implantation and placental pregnancy [46,47]. In mammals, several mechanisms based on the extensive crosstalk between the embryonic trophoblast layer and maternal uterine immune cells prevent the rejection of the allogeneic embryo by the maternal immune system. First, trophoblast does not express MHC II, which otherwise would present embryonic antigens to maternal T-helper cells (Th) and initiate an immune response [48,49]. Additionally, the MHC I genes, which present antigens to maternal cytotoxic T cells, are downregulated in the trophoblast [49,50]. Additionally, regulatory T cells (Tregs) recognize fetal antigens via maternal antigen-presenting cells (APCs) and induce tolerance toward the embryo [51].

**Figure 3:**
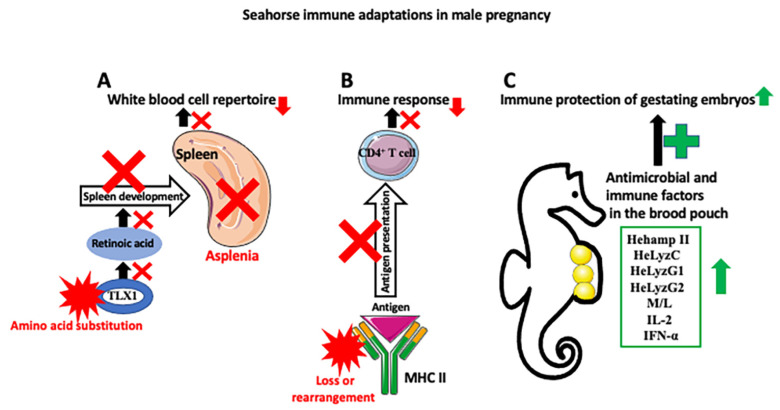
Seahorse immune adaptations in male pregnancy. Three main mechanisms are responsible for the development of seahorse immune tolerance against allogeneic embryos. (**A**) Seahorses are asplenic (spleen loss). Asplenia is caused by the amino acid substitution in the transcription factor TLX1, which, through the retinoic acid pathway, regulates spleen development. Asplenia affects the white blood cell repertoire (causes partial loss of red blood cells, platelets, various subsets of T and B cells, dendritic cells (DCs), and macrophages) and decreases immune response. (**B**) There is a downregulation of the MHC II pathway, either through a loss or rearrangement of MHC II genes. This, in turn, decreases self- and non-self-antigen presentation to the effector immune cells, such as CD4+ T cells, and lowers the immune response against allogenic embryos. (**C**) Despite the weakened immune responses of the parent against embryos, gestating embryos are protected against pathogens abundant in a nonsterile seawater-filled pouch by the increase in antibacterial peptides and immune factors, such as hepcidin (Hehamp II), lysozymes (HeLyzC, HeLyzG1, and HeLyzG2), monocytes and leukocytes, interleukin 2 (IL-2), and interferon alfa (IFN-α) in the pouch. Red X symbol depicts inhibition of disruption of the process, and red star depicts changes in molecule composition or structure.

Thus, the fascinating question is how, during evolution, did the immune system adjust to tolerating the nonself embryo. Because most mammals, except for egg-laying monotremata, such as echidna and platypus, have internal pregnancies, they are useless for reconstructing evolutionary progress in parental immunotolerance. In contrast, male seahorses, with their gradation of pregnancy, from a simple attachment of eggs to the skin, through a different degree of coverage by the skin flaps, to internal gestation within the brood pouch, are perfect for reconstructing evolutionary progress in the development of immune tolerance toward the nonself embryo (Figure 3). Recently, using this approach, Roth et al. [5] studied the immune gene repertoire across male pregnancy gradients in 12 species of seahorses and pipefishes. These detailed studies showed that the evolution of pregnancy coincided with either a complete loss or rearrangement of MHC II pathway genes and correlated with the expansion of the MHC I gene repertoire. MHC II molecules are found on professional antigen-presenting cells such as dendritic cells, mononuclear phagocytes, some endothelial cells, thymic epithelial cells, and B cells, which are crucial for initiating immune response. The antigens presented by the MHC II genes derive from extracellular proteins, while antigens presented by the MHC I genes mainly derive from cytosolic proteins. MHC II binds peptides, which are derived from the proteolysis of self and non-self proteins and presents them to antigen specific CD4+ T cells. On the same theme, studies from another laboratory compared transcriptome-wide gene expression in four syngnathid species with different degrees of paternal involvement at four pregnancy stages (nonpregnant, early, late, and at birth) [52]. They found that the loss or downregulation of MHC pathway gene expression occurs only in species with a brood pouch pregnancy, and that a decrease in MHC pathway gene expression is limited to the early and middle stages of pregnancy. In late pregnancy and at birth, the expression of immune genes was elevated, suggesting that the late embryos, in contrast to the early embryos, are no longer in direct contact with the paternal immune system [52,53].

Comparative genomic analyses across the *Syngnathidae* (pipefishes, seadragons, and seahorses) species showed that the complexity of the immune system gene repertoire decreased in evolution in parallel with the increase in paternal care [54]. This is in startling contrast to a general tendency to increase immune complexity during evolution. The evolutionary increase in immune complexity was partially achieved by the emergence of specialized lymphatic organs such as the spleen. Interestingly, seahorses reduced not only the complexity of the immune genes but also eliminated the spleen during evolution. Liu et al. [54] showed that, in seahorse evolution, the loss of the spleen (asplenia, which causes partial loss of red blood cells, platelets, various subsets of T and B cells, dendritic cells (DCs), and macrophages) is associated with a specific amino-acid replacement in the T-cell leukemia homeobox protein 1 (TLX1, HOX11) transcription factor, which controls spleen development via regulation of retinoic acid metabolism. Tlx1 deletion causes asplenia in mice, and deregulation of TLX1 expression is involved in the pathogenesis of congenital disease in human patients (Figure 3) [55].

The fact that the loss of the spleen and important components of the immune defense does not impair the immunological response of seahorses against the encountered microbes points to the extraordinary flexibility of the vertebrate immune system [5]. The seahorse brood pouch, filled with nutrient-rich and nonsterile seawater, is ideal for bacterial proliferation. So how are embryos protected against these pathogens? Recent studies by Xiao et al. [56] may partially explain how immune protection is achieved in seahorse pregnancy. These studies showed that the seahorse brood pouch contains antimicrobial peptides such as hepcidin, which defend developing embryos against pathogens. Hepcidin is a small cysteine-rich antimicrobial peptide. Vertebrates have two types of hepcidin: hepcidin I (HampI), which regulates iron metabolism, and hepcidin II (HampII), which is present only in fishes and functions in immune defense. Xiao et al. [56] identified four hepcidin II genes (HehampII 1, 2, 3, and 4) in the lined seahorse *Hippocampus erectus*, and followed their expression during successive stages of pregnancy and in the offspring. Although HehampII genes are expressed in all seahorse tissue, the expression increases 20–30 times in the brood pouch placenta and shapely decreases at birth. In the offspring, the expression level of HehampII increases throughout pregnancy and peaks at parturition when newborns are released into the water and encounter a massive number of pathogens [56]. Other molecules which probably participate in the defense against brood pouch pathogens are the antibacterial enzymes of the innate immune system, the lysozymes (Lyz). Lysosomes process the antigen for presentation on MHC molecules and activate Toll-like receptors [57]. Studies of Lyz genes and their expression in the brood pouch of *Hippocampus erectus* identified three different lysozymes: HeLyzC, HeLyzG1, and HeLyzG2 [58]. Comparison of the mRNA expression levels of these three lysozymes in different tissues showed that expression analysis showed that all three HeLyzs were significantly upregulated in the brood pouch but low in gestating embryos and high in neonates. A low level of HELyzs in gestating embryos indicates that while in the brood pouch the embryos are protected by the paternal lysozymes [58]. Studies of overall immune factors such as monocytes/leucocytes (M/L), leucocyte phagocytic rate (LPR), immunoglobulin M (Ig M), interleukin-2 (IL-2), interferon-α (IFN-α), and lysozyme (LZM) in the plasma and epithelial lining of the brood pouch of the lined seahorse *Hippocampus erectus* in different stages of the breeding cycle—pre-pregnancy; early, middle, and late pregnancy; and post-pregnancy—showed the low level of these factors in pre-pregnancy, as well as that the highest level is found in the early and middle pregnancy stages [21].Thus, these results also indicate that the brood pouch epithelium and paternal organism are crucial for the immunoprotection of the offspring (Figure 3).

## 4. Effects of Male Pregnancy on the Microbiome

One of the most fascinating changes occurring during *Syngnathidae* male pregnancy is a profound change in their microbiome. Although there is not much information on this subject in seahorses, there are recent, very detailed studies on pipefish pregnancy [59]. In animals with maternal pregnancy, some microorganisms are transferred to the offspring from the mother, and some colonize newborns (through the mouth) from the environment. In maternal pregnancy, it is very hard to establish if there is any input of microorganisms from the father. Thus, animals with male pregnancy, such as pipefishes or seahorses, are very well suited to study this phenomenon and answer the question of how pregnancy influences the parental microbiome. Recent studies on pipefish *Syngnathus typhle* sequenced microbial 16S rRNA from maternal gonads and the brood pouches of non-pregnant and pregnant males [59]. These studies also assessed the effect of the parental immune system on the complexity of the microbiome. These analyses showed that maternal gonads and male brood pouches contain different microbial species (listed in the paper) and contain different species in early and late pregnancy brood pouches [59]. The most abundant bacteria in the maternal gonads and in the paternal brood pouch were *Marinomonas* (28.0%), *Halomonas* (10.3%), *Aeribacillus* (6.3%), *Ruegeria* (5.8%), *Bacteroidetes* (4.5%), and *Nesterenkonia* (3.8%). Additionally, the paternal immune system changes the bacterial composition to a higher abundance of *Kiloniella*, *Aquimarina*, *Ulvibacter*, and *Marinomonas*. These are commensal bacteria that help fight pathogenic bacteria and possibly boost the immune response in the offspring [59].

## 5. Effects of Environmental Changes and Pollutants on Seahorses

Many species of seahorses are endangered by overfishing, degradation of their habitats, and pollution. Even under the best circumstances, seahorse numbers are low because of the demanding and restrictive lifestyle. Seahorses sparsely inhabit an intricately structured niche, and their survival depends on the elaborate camouflage specific to a particular surrounding. Because seahorses are poor swimmers, nonmigratory, monogamous, and form a lifelong pair bond, finding a new partner when one dies is very difficult. Seahorse reproductive rate, even under optimal conditions, is low because of the small brood size and lengthy parental care. Consequently, seahorses are on the local, international, and IUCN Red List of Threatened Species [60], becoming a bioindicator of crude oil exposure [61]. Studies of the effects of ocean warming and acidification on the physiology and behavior of the long-snouted seahorse *Hippocampus guttulatus*, which has been recently added to list of threatened and/or declining species by the Oslo and Paris (OSPAR) commission, showed that although the adults are quite resistant to ocean warming, the combination of warming and acidification (caused by CO_2_ uptake from the atmosphere) causes lethargy and reduction in feeding and gill ventilation rates [62]. In contrast, the seahorse newborns are much more sensitive to warming, which causes heat-induced hypometabolism [63].

Besides factors related to climate warming, seahorses, like other aquatic animals, are constantly exposed to various types of pollutants. For example, studies of seahorse species inhabiting coastal waters of the Black Sea and China showed a high accumulation of heavy metals (including Cu, Pb, Cd, Cr, and Hg), benzo(a)pyrene (B[a]P), organochlorine pesticides, polycyclic aromatic hydrocarbons (PAHs), and microplastics [64,65,66,67,68].

### 5.1. Microplastics

Current worldwide production has resulted in a staggering 380 million tons of plastic, and meso-, micro-, and nanoplastics formed not only by plastic fragmentation but also released by the laundering of synthetic textiles, and plastic became a major pollutant of aquatic ecosystems [68,69]. Micro- and nanoplastics have similar sizes to the food particles; are often ingested by aquatic organisms; and they can also absorb toxins and heavy metals, which potentiate their harmful effects [70,71]. Studies on microplastic and heavy metals accumulation in eight species of seahorses from the coastal regions of China showed the presence of 92–322 microplastic particles in seahorses’ intestines, which coincided with the accumulation of heavy metals Pb, Cd, and Cr [64,65]. Transcriptomic analysis of the effects of microplastic and heavy metal accumulation on the gene expression of the line seahorse *Hippocampus erectus* showed that heavy metals affected metabolic (protein and fat digestion and absorption, steroid biosynthesis, and glyceride metabolism), immune (ECM–receptor interaction, IgA intestinal immune network, and PI3K-Akt signaling pathway), and apoptotic (iron-dependent programmed cell death) pathways. The microplastic accumulation mainly affected the expression of fatty acid syntheses genes, such as lipid transporter Stard7, an inhibitor of lipoprotein oxidation Apoa4; α-demethylase Cyp51; fatty acyl desaturase Fadsd6; and DNA damage repair genes, such as the post-replicative DNA mismatch repair gene Msh3, ultraviolet light-damaged DNA repair gene Ddb2, multistep DNA repair gene Xrcc2, DNA recombination gene Rad52, oxidative damage DNA repair gene Ogg1, and DNA replication error repair gene Pms2. Both pollutants affected the expression of antioxidant pathway genes and increased the expression of superoxide dismutase (SOD), heat shock proteins 70 and 90 (Hsp70, Hsp90), phosphatidylinositol 3-kinase regulatory subunit (Pik3r1), cytochrome C (Cycs), caspase-9 (Casp9), caspase-3 (Casp3), cyclin-dependent kinase inhibitor (P21), RAC-γ serine/threonine-protein kinase (Akt3), interleukin 10 (IL-10), Toll-like receptor 2 (Tlr2), and chemokine receptor 9 (Ccr9) [65]. This indicates that not only does microplastic by itself have a profound effect on gene expression patterns, but it also exacerbates damage by delivering heavy metals.

### 5.2. Endocrine Disruptors

Among pollutants of aquatic environments and water sources, the endocrine-disrupting chemicals (EDCs) are the most dangerous because, even in low concentrations, they alter sex ratio; affect sexual dimorphism, gonad, and gamete development; and suppress reproductive behavior. They can also cause premature sexual maturation and induce menopause. Additionally, they cause epigenetic modifications of the genome and thus may have multigenerational effects, both in animals and humans [72,73]. One such prevalent EDC is an organotin (organic compound containing toxic heavy metal tin), Tributyltin (TBT). TBT and other organotin anti-fouling compounds (OTCs) are widely used as an anti-fouling paint, which prevents the attachment of water organisms to boats, ships, and fishnets, and serves as a wood and textile preservative and polyvinyl chloride stabilizer. TBT binds to the sediments and suspended materials and can be released into the water over many years [74].

Transcriptome profiling of the effects of environmentally relevant concentrations of TBT on the development of gonads and brood pouch of the lined seahorse (*Hippocampus erectus*) showed that in female seahorses, TBT increased androgen levels, caused atresia of ovarian follicles, zona pellucida breakdown, yolk liquefaction, and hypertrophy of the granulosa cells in the ovaries. It also induced lysosome and autophagosome pathway genes and apoptosis through the suppression of the anti-apoptotic gene FAIM (fas-apoptotic inhibitory molecule) [75]. In males, TBT suppressed cyclic AMP (critical for spermatogenesis and sperm motility), androgen synthesis and spermatogenesis, and caused degeneration of the testes. It also affected angiogenesis, embryos’ nourishment during gestation (by changing transcription of genes of lipids and carbohydrates metabolism pathways in the brood pouch), and upregulated expression of immune response genes in the brood pouch [76]. The effects of TBT on the seahorse immune system, including induction of the antioxidant defense system and stress response genes and severe liver damage, were also confirmed by another study [77].

### 5.3. Antibiotics

Antibiotics present in water have damaging effects on aquatic organisms and diversity of their microbiome [78]. Because of the lack of a spleen and gut-associated lymphatic tissues (GALTs), seahorses are especially vulnerable to any pollutants affecting their microbial diversity and immune response [5]. The gut microbiome health is critical for seahorse well-being and reproduction, and studies showed that male seahorses are very sensitive to changes in the diet before and during pregnancy [79]. These studies showed that although embryo development was mainly supported by maternally supplied yolk, a paternal diet deficient in polyunsaturated fatty acids affected offspring size and elongation, fatty acid composition, and gene expression patterns [79]. Recently, Zhao et al. [80] studied in detail the effect of chronic exposure on two widely used broad-spectrum antibiotics, Triclosan (TCS) and Sulfamethoxazole (SMX), on the transcriptome and microbiome of the *Hippocampus erectus* gut and brood pouch. They showed that these antibiotics affected microbial diversity within the gut and brood pouch and increased the abundance of pathogenic bacteria such as *Legionella*, *Brevibacterium*, *and Staphylococcus*. The antibiotics also caused upregulation of the Toll-like receptors (TLRs), interleukin, chemokine, tumor necrosis factor superfamily, MHC I and MHC II genes, and c-type lectins gene expression in a brood pouch, and affected genes related to metabolic, circadian rhythm, and reproduction pathways in adult and juvenile seahorses [80]. The authors concluded that disruption of microbiome balance exposed the brood pouch to pathogenic bacteria, which triggered inflammation via the TLR and c-type lectin signaling pathways. Increased expression of inflammatory genes and MHCI, whose downregulation is necessary for brood pouch immune tolerance toward the embryos, will be detrimental to seahorse pregnancy.

In summary, in this review, we described features of male pregnancy, brood pouch morphology development, the evolutionary gradation of paternal involvement in egg/embryo caring, and the immunological adaptions required for tolerance of allogeneic embryos. We also reviewed information on hormonal and nonhormonal regulators of male pregnancy and the effects of male pregnancy on the microbiome. Because seahorses are excellent bioindicators of environmental pollutants, we described examples of the effects of microplastics, endocrine disruptors, and antibiotics on seahorse development, their immune system, and their microbiome.

## Data Availability

Not applicable.
